# Video Rain-Streaks Removal by Combining Data-Driven and Feature-Based Models

**DOI:** 10.3390/s21206856

**Published:** 2021-10-15

**Authors:** Muhammad Rafiqul Islam, Manoranjan Paul

**Affiliations:** School of Computing, Mathematics and Engineering, Charles Sturt University, Panorama Ave, Bathurst, NSW 2795, Australia; mpaul@csu.edu.au

**Keywords:** mask RCNN, rain removal, rain-free video, synthetic rain, TA feature

## Abstract

Video analytics and computer vision applications face challenges when using video sequences with low visibility. The visibility of a video sequence is degraded when the sequence is affected by atmospheric interference like rain. Many approaches have been proposed to remove rain streaks from video sequences. Some approaches are based on physical features, and some are based on data-driven (i.e., deep-learning) models. Although the physical features-based approaches have better rain interpretability, the challenges are extracting the appropriate features and fusing them for meaningful rain removal, as the rain streaks and moving objects have dynamic physical characteristics and are difficult to distinguish. Additionally, the outcome of the data-driven models mostly depends on variations relating to the training dataset. It is difficult to include datasets with all possible variations in model training. This paper addresses both issues and proposes a novel hybrid technique where we extract novel physical features and data-driven features and then combine them to create an effective rain-streak removal strategy. The performance of the proposed algorithm has been tested in comparison to several relevant and contemporary methods using benchmark datasets. The experimental result shows that the proposed method outperforms the other methods in terms of subjective, objective, and object detection comparisons for both synthetic and real rain scenarios by removing rain streaks and retaining the moving objects more effectively.

## 1. Introduction

Challenging atmospheric conditions such as rain and snow degrade the visibility of video sequences [1,2,3,4,5,6]. As a result, video analytics and computer vision applications suffer from the degradation of visibility of video sequences, as most algorithms assume a clear, rain-free video sequence [5]. Improving visibility of the video sequences by removing rain streaks has thus become an obligatory processing step for object detection and tracking [7], scene analysis [8], and person reidentification [9]. These tasks have extensive applications such as driverless cars, advanced driver assistant systems, intelligent traffic surveillance systems, security surveillance systems, etc. [8,10,11]. In addition to computer vision applications, the degradation of visibility by rain streaks can also affect the performance of many multimedia processing systems, e.g., content-based image retrieval [12], and image enhancement methods [13]. Therefore, as an important research topic, removing rain streaks and improving the visibility of a video sequence has attracted much attention in recent years in the fields of multimedia, image processing, computer vision, and pattern recognition [2,11,14,15,16].

Rain-streaks removal research can be categorised into two cases, single image-based rain-streaks removal and video-based rain-streaks removal. The methodologies have been designed for both cases. In recent years, various methods have been proposed for rain-streaks removal for both cases: video and single image as the video sequence is a combination of sequential frames (i.e., images) [17,18,19,20,21,22,23]. This paper will concentrate on video-based rain-streaks removal.

The approaches that have been proposed for Rain-Streaks Removal in Video (RSRV) can be categorised into two types. Conventional approaches mainly focus on the apposite implementation of the physical properties of the rain streaks and the prior knowledge of the background scene in the time domain and frequency domain. These methods are used to encode all the exploited features into an optimisation problem and propose algorithms to solve it rationally. In the rest of the paper, we will mention these approaches as a feature-based model. Recently, emerging approaches have been introduced based on data-driven processes, e.g., deep learning. They are mainly based on the specific network architecture using the knowledge of the previously processed relationship of images/videos with and without rain.

Garg and Nayar [24] developed an RSRV method where they proposed that two camera properties, exposure time and depth of the field adjustment, could reduce or even remove the effects of rain in a video sequence. Subsequently, many feature-based approaches have been recommended for the RSRV method and have achieved good results in rain-streak removal with various rain conditions. Wide-ranging primary video-based methods are reviewed in [25]. Video sequences with active scenes that include different objects and motions have been studied in [26]. Kim et al. [27] have focused on the time-domain characteristic of rain streaks and the low-rank characteristic of rain-free videos. Santhaseelan et al. [28] marked and eliminated rain streaks based on phase congruency features. You et al. [29] worked with environments where raindrops were situated on window glass or a car windscreen. In [30], the authors focused on the directional property to propose a tensor-based RSRV method. Ren et al. [31] worked with snow and rain conditions and considered the matrix decomposition technique. Wei et al. [21] have stochastically modelled rain streaks and have not considered the deterministic features. The rain-free background has been modelled using a mix of Gaussians, while Li et al. [32] introduced multiscale convolutional filters from rain data. They have applied a multiscale, convolutional, sparse coding technique to develop the filter. A video may have different kinds of objects and motions, which create difficulties in feature-based models. The challenge is exploiting appropriate features, encoding them into an optimisation problem, and fusing them with a rational algorithm for a meaningful rain removal strategy as rain streaks and objects have dynamic characteristics. As a result, some methods perform better by retaining entire objects but fail to remove some rain streaks. On the other hand, some methods successfully remove more rain streaks but fail to retain the entire object area. Moreover, we observed that too many features may cause misclassification of rain and/or moving object as the fusing of them creates inappropriate results for different kinds of rain.

Recently some existing methods have often used a data-driven approach by designing specific network architectures and precollecting rain-free image/video pairs to learn network parameters to attain complex rain removal functions [33,34]. Later these functions will be used to generate rain-free images/videos. A data-driven model based on a deep-learning network for the RSRV has started to reveal its effectiveness [4,35,36,37,38]. Most of the existing models have been developed based on single-image features but can also be applied to video sequences. These models have mostly addressed the interference/difficulties in visibility caused by the accumulation of rain streaks. In [39], the authors use binary mapping representing rain and without rain pixels to train the rain model. In the rain removal method, they used a contextualised dilated network. A Generative Adversarial Network (GAN)-based deraining method has been proposed in [1]. The authors have also introduced residual learning to develop a rain removal model. A two-stage Recurrent Neural Network (RNN) architecture has also been proposed for video deraining [37]. A sequential deep unrolling framework has been proposed to exploit spatial and temporal features for video deraining [38]. The data-driven models are highly dependent on the pattern or characteristics of the training dataset, where sometimes it is difficult to include all types of rain, objects, and other environmental characteristics for the learning phase. Moreover, most of the existing data-driven methods have targeted certain insightful aspects of rain removal and are advantageous for specific circumstances. In addition to these, sometimes it is difficult to obtain the corresponding videos with rain and without rain for training the deep-learning models. This may cause inadequate model learning; thus, the performance of the deep-learning-based techniques may not at the expected level.

This paper addresses the issues mentioned above, raised by both the approaches, data-driven and feature-based models. First, we consider a single but powerful novel feature, the temporal appearance (TA) of the rain streaks. We observed that normally rain streaks do not appear in a particular pixel position for more than a few adjacent frames. We exploited this property by defining the TA feature to distinguish rain streaks from moving object areas. However, some portions of the moving object areas have been missed out in the generated rain-free frame if we solely depend on the TA property for rain removal. The TA property mainly exploited the brief appearance of a rain streak in a pixel location among adjacent frames. Although the exploitation of this characteristic successfully distinguishes most of the rain streaks, unfortunately, it classifies the brief appearance portion of a moving object as rain. The preliminary idea is published in a conference paper [10].

**Figure 1:**
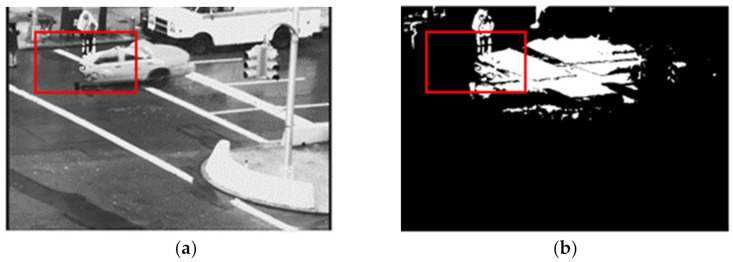
The output of the temporal appearance (TA) feature-based model of the video sequence “Traffic” where some object areas are missing in the rain-free images (red rectangle). (**a**) Generated rain-free image of frame 85 and (**b**) Objects mask of frame 85.

Figure 1 shows the issue mentioned above for TA feature where the background modelling is used to separate both rain and moving regions from the background scene. It shows a frame without rain using the TA feature where the recommended threshold is used. However, sometimes selecting the threshold is difficult for different types of rain. It can lead to keeping an object with less removal of rain or removing rain by missing out portions of object areas. Here, we observe that some pixels of object areas are detected as false-negative, i.e., the object areas are classified as rain streaks and as a result, are missed out in the generated rain-free frame. The object areas need to be detected with pixelwise accuracy to overcome this problem and obtain distortion-free objects in a complete rain-free frame. Moreover, the TA property also depends on the frame rate; thus, an adaptive TA feature-based technique is necessary so that TA feature-based technique can effectively work with different frame rates.

Mask R-CNN is a data-driven model which is used to segment and construct a pixelwise mask for each object in an image. The Mask R-CNN algorithm was introduced by He et al. [40] on top of the Faster R-CNN object detection algorithm [41]. Mask R-CNN provides better object segmentation performance over feature-based methods. It is suitable and superior in specific occasions and inconsistent in many other occasions as a data-driven model. Because it is difficult or sometimes impossible to include all the existing occasions in the training dataset, another issue in the Mask R-CNN model is that it segments every object in an image, whether the object is dynamic or static. This is not required for many applications, including the rain removal process. To overcome this problem, we propose a hybrid method by combining both data-driven and feature-based models where the rain streaks identified by an adaptive (i.e., frame-rate invariant) TA property will be refined by the object mask identified by Mask R-CNN so that the moving object areas are no longer identified as rain streaks.

This paper proposes a novel hybrid technique to combine data-driven and feature-based models for better rain removal without sacrificing the quality of moving objects. The proposed algorithm fuses the prediction’s data from three sources: the Mask R-CNN model, an adaptive TA feature, and the background and foreground extraction in two separate steps. The three predicted data are combined to generate a rain-free frame. To make the proposed method effective at different frame rates, we modify the TA feature by an adaptive threshold to work in different frame rates as the duration of rain appearance in terms of frame number depends on the frame rate. The main contributions of the paper can be summarised as:

- We introduce and formulate a novel hybrid technique to combine data-driven and feature-based models to overcome individual limitations.
- We develop a pixelwise segmentation strategy to distinguish between rain, moving objects and background pixels for fine-level accuracy to remove rain streaks by keeping the entire area of the moving objects.
- For better rain interpretability, we exploit outcomes from the deep-learning-based model with the physical-feature-based technique.
- We propose and formulate TA features of the rain streaks with an adaptive threshold to separate them from the moving objects irrespective of the frame rate.

The rest of the paper is organized as follows: Section 2 explains the proposed method, where all the steps are discussed with justifications. Section 3 provides the experimental setup and analysis of the results and Section 4 concludes the paper.

## 2. Materials and Methods

**Figure 2:**
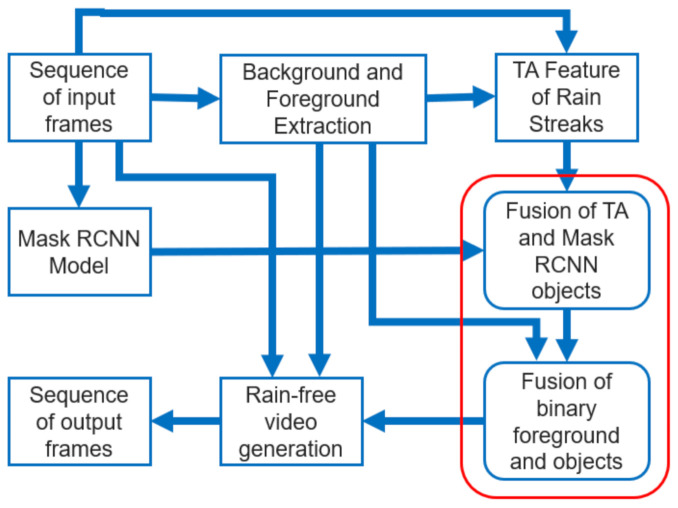
Block diagram of the proposed method.

We combined the feature-based and data-driven models to remove rain streaks from a video sequence and generate a rain-free video. Figure 2 shows the steps executed in the proposed method. Here, we used the Mask R-CNN model and adaptive TA feature-based model to detect the objects in a video sequence. Then, we fused the predicted object areas by the Mask R-CNN model and predicted object areas by the adaptive TA feature-based model. This fusion then predicted only the moving objects and eliminated the static objects from the Mask R-CNN prediction. In the next step, we again fused the binary foreground data with the predicted data of the previous step to finalise the mask area of the moving objects. All the vital steps are discussed in detail in the following subsections. Note that here input frames are in YCbCr colour format and the processes described below are applied on the Y component of the input frame.

### 2.1. Background and Foreground Extraction

Many dynamic background modelling approaches [42,43,44] are available in the literature. Some of them are statistical or nonstatistical [45]. Some complex models have been proposed for better accuracy, e.g., a mixture of Gaussian (MoG) [46,47] and Spatiotemporal scheme-based models [48]. The basic concept for developing these models is similar. The background remains the same over all the frames in a video scene captured by a static camera, except for the interference of moving objects and change of light. Thus, this background layer can be formulated as recovering a low-dimensional subspace [49,50,51,52,53]. The regular approach to subspace learning is the subsequent low-rank matrix factorisation (LRMF):

(1) $$B=Fold\;\left(UV^{T}\right)$$

where, $U\in R^{d\times r}\;\mathrm{is}\;\mathrm{mixing}\;\mathrm{matrix}$, $V\in R^{n\times r}$ is encoding matrix; $r<\min\left(d,n\right)$, and the operation of *‘Fold*’ refers to the foldup of each column of a matrix into the corresponding frame matrix of a tensor. The superscript *T* indicates the transpose of a matrix. Here, *B* is the data matrix, and *d* and *n* are the column and row of the images, respectively.

At each frame, we generate a background frame. We use the background frame to find rain streaks and the moving objects, in order to generate the rain-free video in the proposed method.

**Figure 3:**
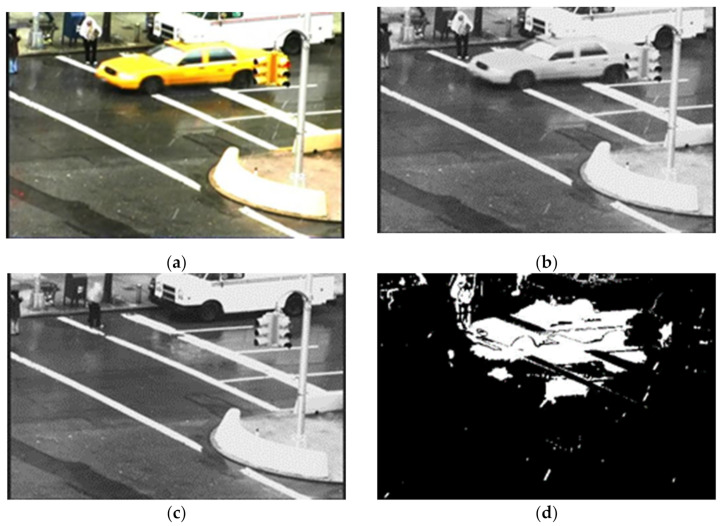
Results of background modelling of the “Traffic” video sequence to demonstrate the separation of moving regions, including rain streaks from the background (**a**) input frame 79 (**b**) Y component of input (**c**) background of the frame 79 and (**d**) binary foreground of the frame 79.

Initially, we generate the foreground by subtracting the background from the input frame, and then we use an intensity threshold to generate the binary image of the foreground.

(2) $$F_{n}=\left\{\begin{array}{c} 1,\;\;\left|I_{n}-B_{n}\right|>Threshold\\ 0,\;\;\;\;\;\;\;\;\;\;\;\;\;\;\;\;\;\;\;\;\;\;\;otherwise \end{array}\right.$$

where *F_n_* is a foreground binary image of the *n*th frame, *I_n_* is the input *n*th frame and *B_n_* is the background frame at the *n*th frame. Here, we use an intensity threshold value of 20 to eliminate the effect of other light or illumination interference from the generated foreground binary image [42,54,55]. This image contains rain streaks and moving objects. Figure 3 shows the outcome of background and foreground extraction.

### 2.2. TA Feature-Based Model

After applying the background extraction, we separate the background and binary foreground of the current frame. This binary foreground contains rain streaks and other moving objects, where both are dynamic and have different characteristics. We exploited the TA feature of rain streaks to separate the rain streaks and other moving objects. It was observed that the rain streaks appear in a location discreetly, while the movement of moving objects is smooth and consistent. The duration of the appearance of rain streaks in a location is only for a few frames based on the frame rate. We developed the TA feature-based model to separate the moving objects and rain streaks based on this characteristic of rain streaks.

**Figure 4:**
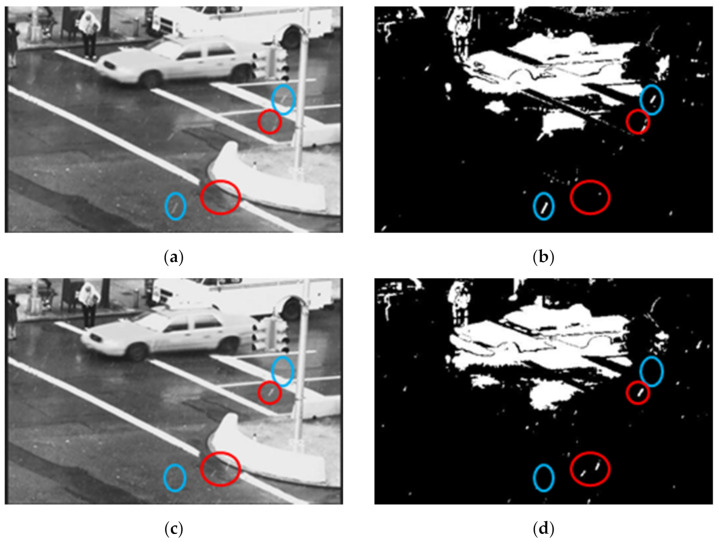
Observation of the temporal appearance property of the rain streaks in the “Traffic” video sequence. Blue and red circles show the appearance and disappearance of rain streaks in the same position. (**a**) input frame 79 (**b**) binary foreground of frame 79 (**c**) input frame 80 and (**d**) binary foreground of frame 80.

Figure 4 demonstrates the TA property of rain streaks. Two adjacent frames (Frame 79 and Frame 80) of the video sequence “Traffic” represent rain streaks in four locations of each frame. Rain streaks appear at two blue circles in Frame 79 but disappear in Frame 80. Rain streaks do not appear in the two red circles in Frame 79 but appear in Frame 80. This observation demonstrates that the rain streaks appear at a particular location of a frame in a video for a brief time and may comprise one or a few frames depending on the frame rate of the capturing device. However, the moving objects usually do not show a discreet appearance characteristic like rain streaks in an area. Rain streaks appear in a video discreetly; they frequently change location for low- to mid-intensity rain. In comparison, moving objects change location smoothly and consistently (see the moving car in Figure 4).

**Figure 5:**
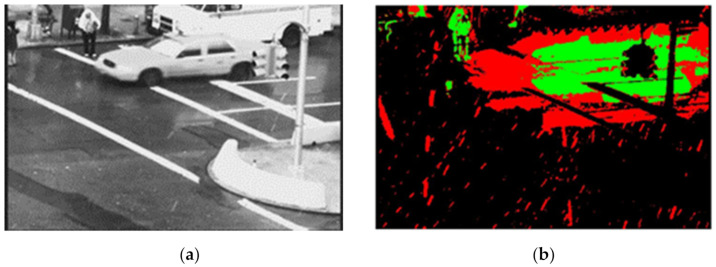
(**a**) Input frame 79 and (**b**) Mask of 79th frame represents background, rain streaks and moving objects of video sequence “Traffic” where black, green and red colours represent background, object, and rain streaks areas, respectively.

We apply the TA characteristic of rain streaks in a modified form to separate rain streaks from the moving objects of the binary foreground. A mask is generated using the binary image *F* for each frame against its adjacent previous *m* number of frames to model rain streaks and analyse the temporal feature. In the binary image, ‘1’ represents the binary foreground comprising rain and other moving objects, and ‘0’ represents the background. TA object mask is predicted based on the following equations:

(3) $$M_{n}=\overset{n-m}{\underset{i=n}{\sum }}F_{i};\left(i=n,n-1,n-2,\ldots n-m\right)$$

(4) $$Obj_{TA}=\left\{\begin{array}{c} 1,\;\;M\geq D_{th}\;\\ 0,\;\;M<D_{th}\;\; \end{array}\right.\;\;where,\;\;D_{th}=c\times f_{r}$$

where *M* represents a mask of the *nth* frame, which contains the foreground’s appearance value, *F* represents the binary foreground of adjacent frames (generated by equation (2)), and *m* is the number of adjacent previous frames considered. The previous *m* number of frames is used to make the decision contemporary, as the scene may be changing significantly enough. In Equation (4), *Obj_TA_* is the predicted object mask of the TA model, *D_th_* denotes duration threshold, *c* is duration threshold coefficient and *f_r_* denotes frame rate of the video sequence. The mask may not be relevant to represent the recent changes. We consider every pixel location’s appearance value in the mask *M*. If the appearance value is more than a certain duration threshold in terms of the frame rate of the video, it is considered as the part of the object area and any value more than zero and up to that duration threshold is considered as the rain area; otherwise, it is considered as part of the background area. We use the duration threshold coefficient *c =* 0.25 or 25% of the frame rate to classify the rain, object, and background areas. The appearance duration varies with the capturing frame rates (see explanation below). In Figure 5, the green area is considered as the object area, the red area as the rain streaks area and the black area as the background area.

**Figure 6:**
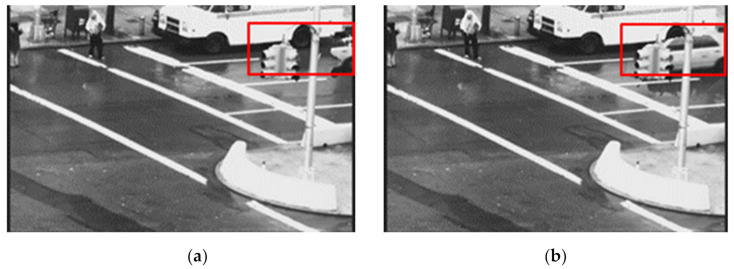
(**a**) The effect (see red rectangle marked area) of the different frame rates on TA output with a constant value of threshold of TA modelling on the “Traffic” video sequence. TA output: (**a**) At frame rate 12 fps and (**b**) At frame rate 24 fps.

The duration threshold *D_th_* mostly depends on the frame rate, because if the capture device is operating at a high frame rate, a rain streak will appear in a greater number of frames. Equation (4) shows the linear property of *D_th_* to the frame rate *f_r_*. This is why for better rain removal, we need to make sure the proposed method is adaptive to the frame rates, or in other words, the proposed method should be applicable for different frame rates. Thus, the duration threshold *D_th_* we have used against mask *M* to predict TA object mask *Obj_TA_* is a function of the frame rates so that the threshold can be adaptive to the frame rate for the success of rain removal. Figure 6 shows the effect of different frame rates of a video if we use a constant value of the threshold in different frame rates. For video with a higher frame rate, the loss of moving objects is less compared to that of a lower frame rate. The results are different for the different frame rates of the video with a fixed threshold. Thus, we can successfully exploit the TA property using an adaptive threshold for different frame rates.

**Figure 7:**
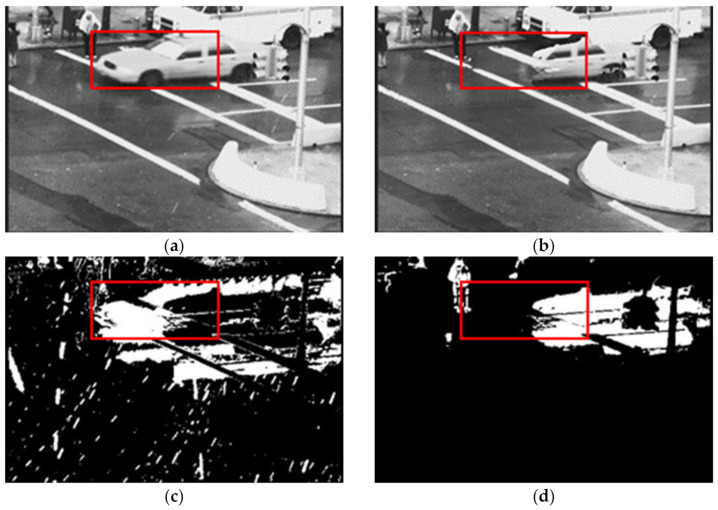
The output of the TA feature-based model of the video sequence “Traffic”. Red rectangle marked area shows indicates the limitation of TA model. (**a**) Input frame 79 (**b**) Generated rain-free image of frame 79 (**c**) Rain streaks of frame 79 and (**d**) TA Objects mask of frame 79.

Figure 7 shows the outcome of the TA feature-based model. Here, we observe that some object areas are classified as rain streaks and missed out in the generated rain-free frame. This distortion of moving objects is not expected in the rain-free image of a video frame. The object area needs to be detected with pixelwise accuracy to overcome the problem and obtain distortion-free objects in a rain-free frame.

### 2.3. Mask R-CNN Model

The Mask R-CNN model is a Deep Neural Network (DNN)-based model. Mask R-CNN is developed on top of the previous object detection model, Faster R-CNN. Faster R-CNN is a region-based convolutional neural network [41]. Mask R-CNN performs object detection with a bounding box and instance segmentation that enables us to obtain a pixelwise mask for each object in an image. Here, we use the model, which is pretrained on the COCO dataset. This dataset includes a total of 80 object classes (plus one background class) that we can detect and segment from an input image. The most common objects are included in the training dataset, whereas rain streaks are not in the training data. Thus, the model detects only objects. Here, we feed the input video frame to the model and obtain Mask R-CNN object mask, which includes a pixelwise mask for each object of the frames in a separate group of pixels.

**Figure 8:**
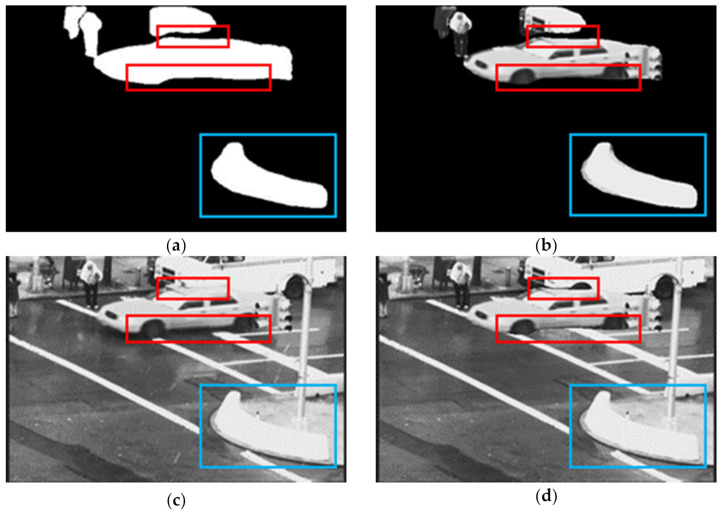
The output of the Mask R-CNN model-based process of the video sequence “Traffic”, frame 79. Red rectangle area indicates missing area of moving objects and the blue rectangle area indicates the false detection of Mask R-CNN. (**a**) Mask R-CNN Object mask (**b**) Segmented objects. (**c**) Input frame 79 and (**d**) Generated rain-free image by Mask R-CNN.

Figure 8 shows the obtained pixelwise mask and segmented objects. The process detects a car, pedestrian and other objects where some of them are not moving objects (blue boxes in Figure 8). The red boxes indicate where the mask of the car misses some parts of it. To recover this distortion and eliminate the static objects, we combine the data-driven model and feature-based model.

### 2.4. Detecting Moving Objects by Fusing Prediction of TA Model and Mask R-CNN Model

The TA and Mask R-CNN models detect objects using a different technique. The TA model separates moving objects and rain streaks from the binary foreground. We have obtained separated TA object masks in an individual binary image.

The mask R-CNN model is a deep-learning technique that makes each object’s pixelwise mask (dynamic and static). This mask enables us to segment the object area from the current frame.

**Figure 9:**
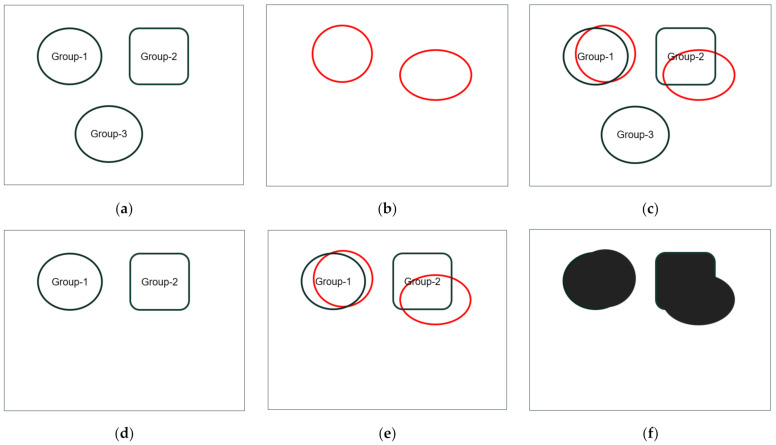
Details of the proposed fusion process. (**a**) Data of model 1 (**b**) Data of model 2 (**c**) Group selection (**d**) Selected groups (**e**) “or” operation and (**f**) Fusion results.

We fuse the predicted object’s pixels from the TA object mask and Mask R-CNN object mask to detect moving objects and eliminate static objects, as the static objects are already in the background frame. Here, pixels of the predicted object in the Mask R-CNN object mask are distributed into a separate group of pixels, and pixels of the predicted objects in the TA objects mask are distributed in a single group of pixels. Figure 9 explains the fusion strategy.

In Figure 9a, model 1 comprises three separate groups for three different objects’ pixels. Model 2 includes two different objects’ pixels in a single group (Figure 9b). The fusion strategy is to select groups from model 1 by applying the pixelwise logical “and” operation between each group of model 1 and model 2. If the process found that model 1 and model 2 predict the same pixels (binary 1 after ‘and’ operation at any pixel position), the groups of model 1 including those pixels are selected for prediction. Figure 9c,d shows the process and outcomes of the group selection. Two groups are selected, and one group is eliminated as it has no common pixels with the model 2 pixels’ group. Then, we integrate the obtained result (selected groups of model 1) and model 2 pixels’ group by applying the logical “or” operation to predict the object mask. Figure 9e,f shows the integration process and fusion results.

**Figure 10:**
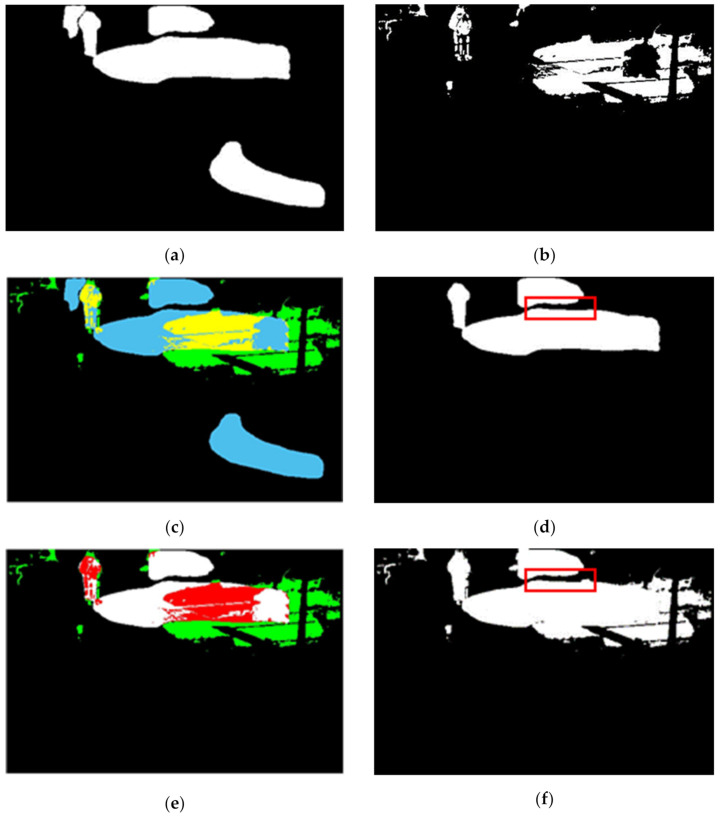
Results after fusing data of the Mask R-CNN model and the TA model for the video sequence “Traffic”, frame 79. Red circle (**a**) Mask R-CNN object mask (**b**) TA object mask (**c**) Predicted object pixels of both models (blue and green) including common pixels (yellow) (**d**) Selected clusters (**e**) Integration of selected clusters and predicted pixels of TA model. Additionally, (**f**) Predicted object mask after fusion.

Figure 10a,b shows the Mask R-CNN object mask and TA object mask, where the Mask R-CNN object mask is assumed as model 1 and the TA object mask as model 2. In Figure 10c, the Mask R-CNN predicted object’s pixels are represented in blue and TA predicted object’s pixels are represented in green. Yellow pixels represent the common pixels of both models. Figure 10d shows the selected three objects (pedestrian, car, and front part of the bus) of the Mask R-CNN object mask, where the pedestrian is selected because the pedestrian has some movement. Then, we integrate the selected objects of the Mask R-CNN object mask with the predicted object’s pixels of the TA object mask and obtain a fusion result. Figure 10e,f shows the integration process and fusion result, respectively. Here, the red boxes indicate that the mask of the car still misses some part of it. To recover this distortion, we combine the obtained result (predicted object mask) with the binary foreground data of the current frame.

### 2.5. Predicting Mask Area of the Objects by Fusing Binary Foreground and Predicted Object Mask in the Previous Step

**Figure 11:**
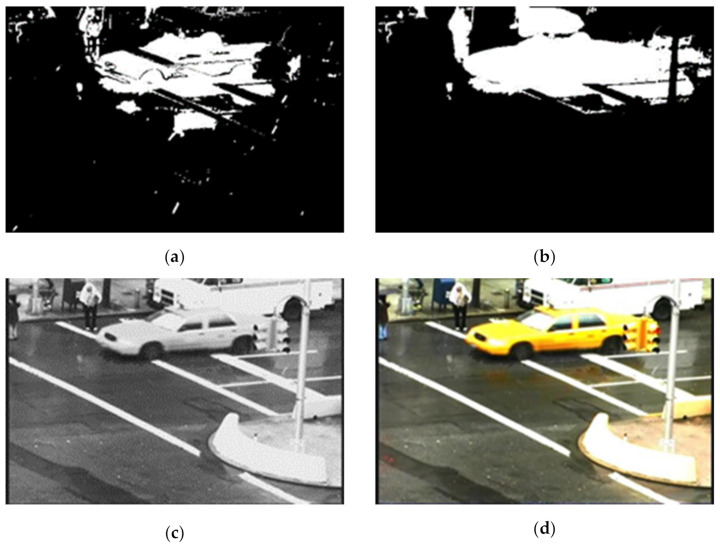
Results after fusing data of the binary foreground and the predicted object mask of previous step for the video sequence “Traffic” frame 79. (**a**) Binary Foreground (**b**) Final object mask (**c**) Generated rain-free frame and (**d**) Final rain-free frame.

The binary foreground extracted from the background and foreground extraction step contains a moving object area and rain streaks typically. We can recover the distorted object area from the binary foreground information. We fuse these two data in such a way that it can generate a less distorted object mask. We divide all the connected pixels of the binary foreground into a different group of pixels. Each group includes connected pixels. Here, we apply the fusion strategy explained in the previous section, where the binary foreground data is model 1, and the predicted object mask from the previous step is model 2. Then, we integrate both the obtained results, applying the logical “or” operation. Figure 11 shows the outcomes of the fusion.

### 2.6. Rain-Free Video Generation

After applying all those extracted features, we have generated an object mask for the current processing frame. We have used both the generated background frame at the current frame position and the current frame to generate a rain-free video frame. For example, we use both the *i*th background and the *i*th frame to generate the rain-free frame for the current *i*th frame. We identify each pixel as a background, rain or moving object through the processes mentioned earlier. The corresponding pixel intensity is taken from the background frame if the pixel is identified as a background or as rain for a rain-free frame. If the pixel is identified as a moving object, then the corresponding pixel intensity is taken from the current frame. Figure 11c,d shows the final generated rain-free image.

## 3. Results

We have conducted experiments using video sequences with real rain to compare the performance of the proposed method and other contemporary and relevant methods. This comparison provides a subjective quality assessment, as there is no ground truth for the rain-free real videos. We also compare the performance using video sequences with synthetic rain to understand subjective and objective measurements, as the synthetic video sequences have ground truth. We have tested the proposed method on a benchmark dataset called the CDNET dataset [56], which includes video sequences with both real and synthetic rain. All the video sequences are captured in different situations. Some have dynamic objects and others do not have any dynamic objects. We have considered regular rainy videos, some include heavy rain, some moderate rain, and some light rain. We have considered four existing methods to compare the performance of the proposed method including three model-based video deraining methods, PMOG [21], MS-CSC [32], and TA [10] and one network architecture-based image deraining method, CGAN [57]. These methods are relatively recent and are relevant to the proposed method.

### 3.1. Real-Rain Video Sequences

**Figure 12:**
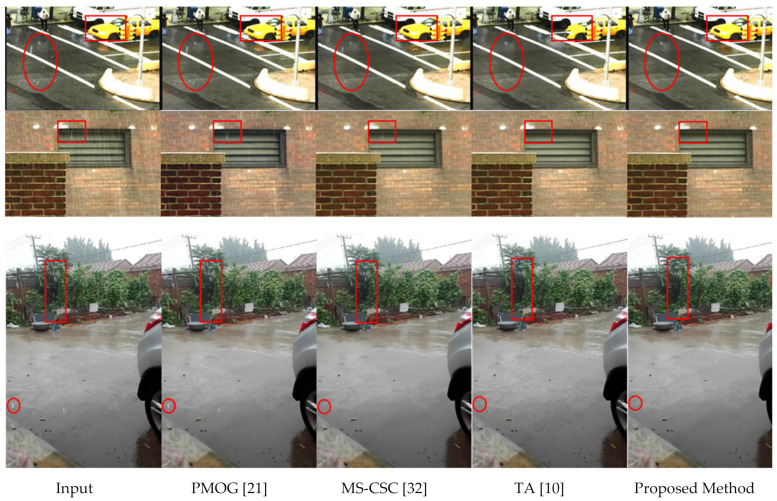
Comparison of the results of different methods (see the red circle and rectangle area closely) for the real-rain video sequences “Traffic” (1st row), “Wall” (2nd row), and “Yard” (3rd row), respectively.

The first row of Figure 12 shows a comparison of the experimental results of frame 72 of the video sequence “traffic”. This video sequence includes a moving car and a pedestrian waiting to cross a road in light rain. The experimental results clearly show that the proposed method outperforms these contemporary methods. The red circle and box show the proposed method removes more rain streaks with no object distortion.

The second and third rows compare the video sequences “Wall” and “Yard” experimental results. They do not include moving objects. The proposed method performs better than the other methods. It has removed more rain streaks than the other methods. The rectangle and circle marked areas clearly show that the proposed method can remove more rain streaks.

### 3.2. Synthetic-Rain Video Sequences

**Figure 13:**
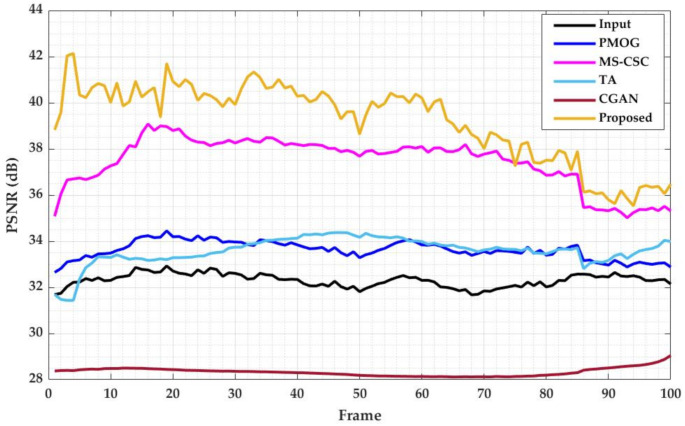
Quantitative comparison of the proposed method with relevant methods in terms of PSNR value.

Figure 13 shows a quantitative comparison of the proposed method against the other methods using the video sequence “Truck”, focusing on the PSNR value in each frame. In this figure, the input PSNRs mean the PSNRs of the input frames (i.e., synthesized rainy frames) against ground truth frames (i.e., without rain) should be the lowest as they have rain. However, CGAN’s [57] PSNR value is the lowest in the graph for every frame. This method degrades the image quality in the rain-free image by blurring the image and losing some information from the background or foreground. This is evidence of the limitation of the data-driven method, dependent on the training dataset. The curve shows the PSNR value falls steeply towards the end for the other three methods. This is mainly due to the content of the video and the amount of rain. In the beginning, the object appears smaller due to the camera position. The amount of rain is also lower in the area where the object is located. However, gradually the object appears bigger and moves to the area where the amount of rain is larger, including the amount of rain in the area of moving objects. Thus, the algorithms show less PSNR towards the end. The proposed method outperforms all methods for all frames except few frames compared to MS-CSC [32]. This demonstrates that the proposed method successfully removes rain from the frames and retains the moving object with better quality.

**Figure 14:**
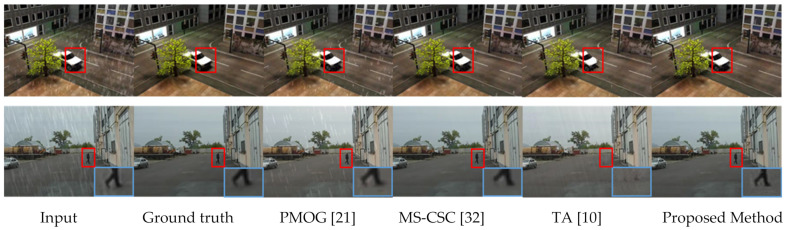
Comparison of the results of different methods (see the red and blue rectangle area closely) for the synthetic-rain video sequences “Truck” (1st row), and “Park” (2nd row), respectively.

The first row of Figure 14 shows the results of a synthetic video sequence called “Truck” at frame 60. The proposed method can remove almost all rain streaks while the other methods fail to remove rain streaks in several areas. The red box shows that some distortion occurred in TA method [10].

The second row of Figure 14 shows the results of another video sequence called “Park” at frame 124. The results show that the proposed method and MS-CSC [32] perform very well in rain removal, whereas PMOG [21] is not as good as the proposed method. The blue box areas show that some distortions have occurred in the results of MS-CSC’s [32] method. This portion is a part of a moving man’s leg. The red box shows that the TA [10] output misses the walking man. Moreover, the proposed method successfully removes more rain streaks compared to the other methods by retaining a better quality for moving regions.

### 3.3. Evaluation of User Application

**Figure 15:**
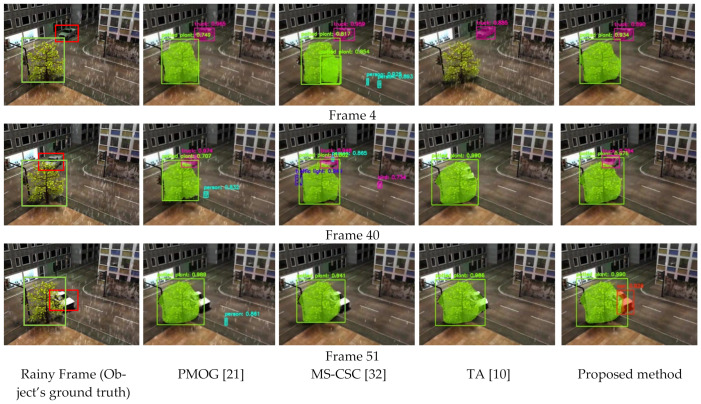
Comparative evaluation of rain-free video for different methods, using the object detection algorithm.

One of the main purposes of rain removal techniques is to detect and recognise different objects, as sometimes object detection/recognition is problematic in a rainy video. Thus, to understand the strength of the proposed rain removal method, we evaluate its performance in terms of object detection/recognition using its rain-free images. Figure 15 shows a comparative analysis of the object detection/recognition algorithms’ results on rain-free video frames generated by the proposed method and the other relevant methods. The comparisons are represented for the video sequence “Truck”. Here, we use the Mask R-CNN model as an object detection/recognition algorithm. Mask R-CNN performs most accurately on the proposed method’s rain-free frames. Figure 15 demonstrates that the objects, i.e., the truck and plant, are correctly detected/recognised from the rain-free images generated by the proposed method. However, the person and bird are wrongly detected/recognised in the rain-free images generated by MS-CSC [32] in frames 4 and 40, and the truck is not detected in frame 51 by the algorithm. For PMOG’s result, the algorithm wrongly detects a person in frames 40 and 51. For the TA [10] result, the algorithm misses one object in all three frames. This is evidence of the superiority of the proposed method against other relevant methods where the rain streaks are successfully removed and the objects are successfully retained in the rain-free images.

Table 1 represents a comparative evaluation of the performance of the object detection algorithms on rain-free video from different methods in two different evaluation metrics: precision and recall. We have calculated the precision and recall based on Figure 15. We have found true positive (TP), False positive (FP) and False Negative (FN) from Figure 15 based on the object detection for all three frames for different methods. A TP is an outcome where the model correctly predicts the positive objects/class. A FP is an outcome where the model incorrectly predicts the positive objects/class. Additionally, a FN is an outcome where the model incorrectly predicts the negative objects/class.

Then, we calculate the precision and recall using the equation below.

(5) Precision=TPTP+FP

(6) Recall=TPTP+FN

The precision and recall values show that the proposed method outperforms the state-of-the-art methods for every frame.

## 4. Conclusions

In this paper, we combined a data-driven model and a feature-based model to address their individual drawbacks. For this, we applied a hybrid technique to combine both models and fuse the models’ predictions. To verify the superiority of the proposed method, we used video sequences with both real and synthetic rains, and compared the performances against four contemporary and relevant methods. The experimental results confirm that the proposed method outperforms those methods by providing better rain-free video and better-quality moving regions. The better rain-free video is demonstrated in terms of better objective and subjective quality comparisons as well as accurate object detection/recognition evidence. Furthermore, as the proposed method used the physical property of the rain and moving objects, it has better interpretability compared to the solely data-driven, i.e., deep-learning, approaches.

## Figures and Tables

**Table 1:** Precision and recall table of object detection algorithm on rain-free video from different methods.

**Precision**				
	**PMOG [21]**	**MS-CSC [32]**	**TA [10]**	**Proposed Method**
Frame 4	1	0.4	1	1
Frame 40	0.67	0.5	1	1
Frame 51	0.5	1	1	1
Average	0.72	0.63	1	1
**Recall**				
	**PMOG [21]**	**MS-CSC [32]**	**TA [10]**	**Proposed Method**
Frame 4	1	1	0.5	1
Frame 40	1	1	0.5	1
Frame 50	0.5	0.5	0.5	1
Average	0.83	0.83	0.5	1

## Data Availability

Not applicable.
